# What Is the Best Practice Method for Quantifying the Health and Economic Benefits of Active Transport?

**DOI:** 10.3390/ijerph17176186

**Published:** 2020-08-26

**Authors:** Holger Möller, Fiona Haigh, Rema Hayek, Lennert Veerman

**Affiliations:** 1 Injury Division, The George Institute for Global Health, Newtown, NSW 2042, Australia; 2 School of Public Health & Community Medicine, UNSW, Sydney, NSW 2052, Australia; 3 Centre for Health Equity Training Research and Evaluation (CHETRE), School of Public Health & Community Medicine, UNSW, Sydney, NSW 2052, Australia; f.haigh@unsw.edu.au; 4 Health Equity Research Development Unit (HERDU), School of Public Health & Community Medicine, UNSW, Sydney, NSW 2052, Australia; 5 Ingham Institute for Applied Medical Research, Liverpool, NSW 2170, Australia; 6 NSW Ministry of Health, St Leonards, NSW 2065, Australia; Rema.Hayek@health.nsw.gov.au; 7 School of Medicine, Gold Coast campus, Griffith University, Southport, QLD 422, Australia; l.veerman@griffith.edu.au

**Keywords:** active transport, walking, cycling, health-benefits, cost-benefits

## Abstract

The aim of this study was to identify a best practice method to cost the health benefits of active transport for use in infrastructure planning in New South Wales, Australia. We systematically reviewed the international literature covering the concept areas of active transport and cost and health benefits. Original publications describing a method to cost the health benefits of active transport, published in 2000–2019 were included. Studies meeting the inclusion criteria were assessed against criteria identified in interviews with key government stakeholders. A total of 2993 studies were identified, 53 were assessed for eligibility, and 19 were included in the review. The most commonly studied active transport modes were cycling (*n* = 8) and walking and cycling (*n* = 6). Exposures considered were physical activity, road transport related injuries and air pollution. The most often applied economic evaluation method was cost benefit analysis (*n* = 8), and costs were commonly calculated by monetising health outcomes. Based on evaluation of models against the criteria, a Multistate Life Table model was recommended as the best method currently available. There is strong and increasing interest in quantifying and costing the health benefits of active transport internationally. Incorporating health-related economic benefits into existing regulatory processes such as cost benefit analyses could provide an effective way to encourage the non-health sector to include health impacts in infrastructure measures.

## 1. Introduction

Physical inactivity is a leading contributor to the rise in non-communicable diseases (NCD) worldwide [1,2], and is estimated to cause around 5% of the overall burden of disease globally [3]. 

In Australia, like many other developed countries, the majority of the population do not meet the recommended daily physical activity guidelines, and around two thirds of the population are estimated to be overweight or obese [4,5]. Active transport, such as walking and cycling, is increasingly recognised as a very promising means of raising physical activity at a population level and reducing the burden of NCDs [6]. Additionally, active transport has wider health and economic benefits through decreased emissions of air pollutants and noise from car travel, and it has the potential to reduce health inequalities as well as transportation inequities, as it applies universally across population groups [7]. 

Although the health benefits of active transport are well established [8,9,10], they are not routinely considered in infrastructure measures [11]. Like elsewhere, in New South Wales, Australia, major infrastructure measures require cost benefit analysis [12]. A wide variety of methods are available to cost the associated health benefits of active transport, but there is no uniform approach. Internationally, a number of studies have been published on costing the health benefits of active transport [13]. These studies used varying methodological approaches, and the estimated benefit to cost ratios of active transport varied widely from −39:1 to 59:1 [13]. The range of methods and estimated benefits can make it challenging to identify a best practice approach.

Against this background, the NSW Ministry of Health commissioned a study to identify a best practice model to cost the health benefits of active transport for routine use in infrastructure planning in NSW, Australia. Towards this aim, we systematically reviewed the international literature to identify methods to cost the health benefits of active transport and evaluated the methods against a set of criteria derived from consultation of key stakeholders.

The project was guided by a cross-agency advisory group consisting of representatives from six NSW government clusters; Treasury; Transport; Planning, Industry and Environment; Premier and Cabinet; Education; and Health. 

## 2. Materials and Methods

### 2.1. Systematic Review 

We systematically reviewed the international literature to provide an overview and critical review of peer reviewed publications and grey literature on methods used to cost the health benefits of active transport.

### 2.2. Search Strategy and Databases Included 

The search strategy was developed to cover the two broad concept areas of active transport and cost and health benefits (Supplement Tables S1 and S2). This strategy was informed by guidelines for systematic reviews [14]; previous systematic reviews on related topics [13,15]; consultation with a research librarian and the NSW Ministry of Health and review of titles, keywords and abstracts of relevant articles known to the authors. The search strategy was tested in PubMed and Scopus. Alternative search strategies covering more narrowly defined concept areas (active transport, cost, health benefits and models/methods) were tested but disregarded in favour of a broader search strategy to maximise the chance that all relevant publications were captured.

The full electronic database search covered 14 databases from the health, social science, leisure, transport, economic and built environment domains. (Supplement Table S2).

A search of the grey literature was carried out by searching reference lists of selected studies, tracking citing documents and searching the internet using Google. Google searches used the advanced search function. A combination of terms from Table 1 was used, and the first 100 titles were scanned for relevance using the study inclusion criteria. 

### 2.3. Inclusion Criteria, Data Extraction and Quality Appraisal

The database search was carried out by two researchers (H.M. and A.M.). Inclusion criteria were:

1. Be published in English between 1 January 2000 and April 2019.
2. Be in the public domain, either as academic papers in peer reviewed journals or studies from the “grey” literature such as government reports and commissioned documents.
3. Be a primary study. Reviews and commentaries were excluded.
4a. Present a model that can be used for economic evaluation of active transport. Applications of already established models were not included unless they represented an extension of the method.
4b. Reproducible in a different setting.
5. Study conducted for the Australian context, or that of other high-income countries.
6. All age groups were considered.

Where it was unclear whether articles met the inclusion from the abstract and title, full text articles were sourced. Full text analysis and data extraction was carried out by the lead author. Two researchers (A.B. and S.S.) independently analysed a subsample of 20% each, compared results and discussed inconsistencies. Disagreement was resolved through discussion among the research team. 

Studies meeting the inclusion criteria were summarised using a standardised data extraction form (Supplement Table S3).

### 2.4. Quality Assessment/Rating of Method 

The methodological quality of studies was assessed using the Consolidated Health Economic Evaluation Reporting Standards (CHEERS) checklist [16] (Supplement Table S4) as well as a list of criteria, identified in semi-structured interviews with key stakeholders (Table 1). Only evaluation of studies against criteria from stakeholder consultation are shown in the main document, because these were specifically tailored to the requirements of a best practice method to cost the health benefits of active transport. In comparison, the CHEERS criteria were found not to be sufficiently specific for evaluation of the quality studies identified in our review. 

Stakeholder mapping was carried out with input from the cross-agency advisory group (advisory group) and further stakeholders were identified during the consultations. Eleven interviews and one focus group were carried out. The interviews identified the current approaches used by public sector agencies in NSW to cost the health benefits of active transport, organisational readiness and institutionalisation, and the requirements for a method to be integrated into cost benefit analysis (CBA) of infrastructure projects. The information gathered during the consultation was used to develop criteria to rate models identified in the systematic review and to benchmark NSW practice against international best practice (Table 1). As a minimum, the included health outcomes associated with physical activity should be breast cancer, colon cancer, ischemic stroke, ischemic heart disease and type 2 diabetes, and those with air pollution ischemic stroke, ischemic heart disease, tracheal, bronchus and lung cancer, and chronic obstructive pulmonary disease. The selection of health outcomes was informed by the evidence from the Global Burden of Disease study [3]. For comparison and model ranking assessment measures were assessed in a dichotomous fashion (Yes/No).

## 3. Results

### 3.1. Literature Review

**Figure 1:**
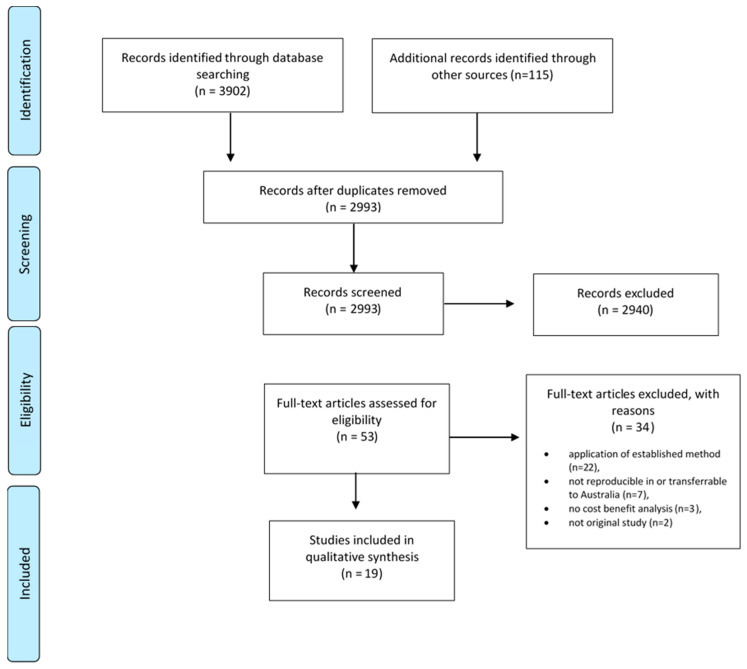
PRISMA diagram flow chart of systematic literature search [33].

The database search identified 3902 articles. An additional 115 articles were identified via Google Scholar and the grey literature search (Figure 1). A total of 2993 records remained after removing duplicates. After screening of title and abstract, 2940 records were excluded and 53 remained for full text analysis. A further 34 studies were excluded with reason after full text analysis (Figure 1, Supplement Table S5) leaving a total of 19 studies [8,9,10,17,18,19,20,21,22,23,24,25,26,27,28,29,30,31,32] for data extraction.

### 3.2. Characteristics of Studies Included

Of the 19 studies included for data extraction, most were published in the last decade (*n* = 17) [8,9,10,17,18,19,20,21,23,24,25,26,27,28,31,32] and originated in Europe (*n* = 11) [9,10,17,19,21,24,25,27,28,29,31], New Zealand and Australia (*n* = 6) [8,18,20,22,26,32] and the United States (US) (*n* = 2) [23,30] (Table 2). The most commonly studied active transport mode was cycling (*n* = 8) [17,21,23,24,25,26,28,31], followed by walking and cycling (*n* = 6) [8,10,18,19,22,29] and walking (*n* = 2) [30,32]. The remaining three studies looked at physical activity in general [9,20,27].

#### 3.2.1. Statistical Models

The most commonly used statistical models to estimate the health impacts of active transport were burden of disease (BoD) methodology (*n* = 12) [9,10,17,19,21,22,24,25,27,28,31,32] and multistate life table methods (Supplement Box 1) [8,18,20]. One study used a Markov model [23] and one system dynamics modelling [26]. Two studies did not calculate health outcomes but applied values derived from other studies to the proportion of those meeting activity targets after the intervention [29,30]. 

#### 3.2.2. Exposures and Health Outcomes 

Exposures considered in the studies in relation to active transport and health outcomes were physical activity, road transport related injuries and air pollution. Most studies included all three exposures (*n* = 10) [8,9,19,21,23,24,26,27,28], five studies considered physical activity only [20,22,29,30,32], three studies physical activity and road transport injuries [17,18,31], and one study only included air pollution [25]. The health outcomes included for the different exposures varied between studies. For physical activity commonly included outcomes were all-cause mortality, cardiovascular disease, colon cancer, breast cancer, diabetes, dementia and depression. One study focused on obesity related health outcomes ischaemic heart disease, hypertensive heart disease, ischaemic stroke, diabetes, colorectal cancer, kidney cancer, breast cancer, endometrial cancer and osteoarthritis [18] (Table 2). Road transport related injury morbidity and mortality outcomes were usually derived from local registry data. Air pollution outcomes varied between studies and included all-cause mortality, cardio-respiratory diseases, lung cancer, acute respiratory infections, cardiovascular disease, stroke, type 2 diabetes, preterm birth and low birth weight.

#### 3.2.3. Outcome Measures

Health related outcome measures included in the different studies ranged from number of deaths and new cases of disease, Years of Life lived with Disability (YLD), Years of Life Lost (YLL), summary measures of population health, which combine morbidity and mortality outcomes, such as Disability Adjusted Life Years (DALY), Quality Adjusted Life Years (QALY) and Health Adjusted Life Years (HALY) and health care costs. 

#### 3.2.4. Economic Evaluation

The most often applied economic evaluation method was cost benefit analysis (*n* = 8) [8,10,17,19,22,29,30,32] followed by cost effectiveness analysis (*n* = 3) [23,26,31] and cost utility analysis [18,20]. Six studies did not include an economic evaluation but were included in the review because they presented methods to quantify the impact of active travel on health and could be extended for use in economic evaluations [9,21,24,25,27,28].

#### 3.2.5. Costing Health Benefits 

Health related costs were commonly calculated by monetising health outcomes. For deaths, years of life lost or summary measures of population health, this was usually calculated by applying the value of a statistical life (VSL) or the value of a statistical life year (VSLY) to the number of deaths and Disability Adjusted Life Years (DALYS), Quality Adjusted Life Years (QALYs) or Health Adjusted Life Years (HALYs) averted, respectively [8,10,17,19,22]. For models that also considered other health related costs, such as health care costs, the total costs were then calculated as the sum of all health related costs [8]. Two models presented health related costs of active transport as cost per km of active travel by travel mode [19,22].

#### 3.2.6. Discounting 

Most studies did not apply a discount rate in the main analysis [8,9,19,21,22,24,25,26,27,28,30,32]. Studies which applied discounting used 3% [18,20,23,29] and 5% [10,17,31] discount rates.

#### 3.2.7. Modelling of Subgroups and Active Transport Modes

Although only one study presented results disaggregated by population groups [32], most models included in the review allowed for disaggregation [8,9,10,17,18,19,20,21,22,23,24,25,26,27,28,31,32] by modelling population groups separately and adjusting the model input parameters accordingly. Similarly, most models allow for including different types of walking and cycling [8,9,10,17,18,19,20,21,22,23,24,25,26,27,28,31,32], characterised by duration and intensity, and these could be modelled separately [9].

### 3.3. Assessment of Studies 

All models except for three [25,29,30] were evaluated against a set of criteria derived during the stakeholder consultation (Table 3). Two studies were excluded, because they did not model health outcomes but instead applied values derived from other studies to estimate the impact of changes in active travelling behaviour on health [29,30]. A third study [25] was excluded because it only considered air pollution; the minimum requirement identified during stakeholder consultation was for a model was to include physical activity.

The Australian model from Zapata-Diomedi and colleagues [8] was the only one that met all of the selection criteria (Table 2). Its main strength lies in the mathematical model which is based on the proportional multi-cohort multi-state life table Markov model (MSLT). 

## 4. Discussion

A total of 19 studies that presented models to quantify the health-related economic benefits of active transport met the inclusion criteria of the systematic review [8,9,10,17,18,19,20,21,22,23,24,25,26,27,28,29,30,31,32]. Most models were suitable for application to different types of active transport, characterised by duration and intensity of physical activity [8,9,10,17,18,19,20,21,22,23,24,25,26,27,28,31,32] and modelling of population subgroups. Commonly considered exposures in relation to health in the different models were physical activity, air pollution and road transport related injuries. However, studies varied in the exposures and health measures they considered. 

Selection of health measures requires in depth review of the epidemiological evidence. Internationally, the Global Burden of Disease (GBD) study regularly quantifies the burden of disease attributable to risk factors such as physical activity and air pollution among others [3]. As part of this, an international expert working group reviews the evidence on these risk factors on a continuous basis [3]. Evidence gathered in these reviews can be used to inform selection of health outcomes to be included in modelling of active transport. For physical activity, the latest version of the GBD study included the health outcomes breast cancer, colon cancer, diabetes, ischemic stroke and ischemic heart disease [34]. Some studies in this review also included dementia and/or depression as health outcomes associated with physical activity [9,27,28]. For these outcomes, there is no consensus on causality. The lower risk of dementia in physically active people may, at least in part, be attributable to reverse causation: preclinical dementia may lead to reduced activity levels [35]. Concerning depression, a recently published meta-analysis of prospective cohort studies reported protective effect of physical activity on depression in youths, adults and elderly persons [36]. Furthermore, a study from the UK reported positive psychological wellbeing effects associated with active travel [37]. In future models of active transport and health, mental health problems should be considered for inclusion, after critical appraisal of all available evidence. One study in the systematic review focussed on the effect of active transport on obesity [18]. Although a recent review of active transport and obesity found that any potential effect of active transport on body mass index is likely to be relatively small [38], the inclusion of obesity and related health outcomes should be explored in future research. 

For air pollution, a causal relationship of particulate matter with health outcomes ischemic stroke, ischemic heart disease, tracheal, bronchus, lung cancer, chronic obstructive pulmonary disease, lower respiratory tract infection, type 2 diabetes, intracerebral haemorrhage and subarachnoid haemorrhage is commonly accepted [3]. Ischemic stroke, ischemic heart disease, tracheal, bronchus and lung cancer, chronic obstructive pulmonary disease, and lower respiratory tract infection were also considered by the studies in this review that included air pollution. Other health outcomes included for air pollution were preterm birth and low birth weight [27,28]. A meta-analysis of cohort and case control studies showed a relationship between PM_2.5_ and low birth weight and preterm birth [39], but there was large heterogeneity in the included studies, limited control for confounding, and the underlying biological mechanisms are not well understood to date [38]. The inclusion of adverse birth outcomes should be further explored when modelling the health outcomes related to air pollution. 

For road transport injury, studies commonly used routinely collected hospitalisation and mortality data. Although the choice of associated health outcomes for injuries is easier than for physical activity and air pollution, estimating the impact of active transport on injuries can be difficult. To date there is limited evidence on the impact of infrastructure changes or increased uptake of walking and cycling on road transport injuries. Most studies in this review modelled expected road transport injuries by multiplying current injury rates with predicted exposure to walking and cycling due to uptake of active transport. Some incorporated a “safety in numbers” effect, based on evidence suggesting that a motorist is less likely to collide with a person walking and cycling if more people walk or cycle [40]. A small number of serious road transport injuries or fatalities can have a substantial effect on the cost effectiveness of an active transport initiative. For example, a study evaluating the cost-effectives of bicycle lanes in New York found that the probability of injury was the most important variable in the analysis [23]. Future modelling of road transport injuries due to active transport should take this into account, in particular when modelling infrastructure changes such as separated bike lanes. Although costly, these might be highly cost-effective because of reduced road transport injuries, with increased safety enticing more travellers to cycle [23]. 

BoD methodology [41] was the most commonly used approach to model the relationship between active transport and health outcomes [9,10,17,19,21,22,24,25,27,28,31,32]. Five models used more complex statistical approaches such as multistate life table methods, Markov models, and system dynamics modelling [8,18,20,23,26]. The BoD methodology uses the mathematical concept of the population attributable fraction (PAF). The PAF is the proportion of cases for an outcome of interest that can be attributed to a given risk factor among the entire population [42]. Input parameters to calculate the PAF are population exposure to the risk factor and the strength of the association between exposure and health outcome. The burden of disease that could be avoided through elimination of a risk factor (or reaching optimal levels of exposure) can then be calculated by multiplying the PAF by the number of observed and predicted cases for the associated health outcomes in the population. In comparison, the proportional multi-state life table (MSLT) method calculates changes in health outcomes from uptake of active transport by simulating two populations, the population as it is (and is expected to be in future) and an identical population that has been exposed to changes in active transport [43]. The proportional MSLT allows for the inclusion of multiple health conditions whilst allowing for comorbidities. Compared with BoD methodology, MSLT methods have the potential to generate more accurate estimates by modelling the effect of risk factor over a lifetime and taking multiple diseases and comorbidities into account. Future models of active transport should therefore use more complex mathematical models such a MSLT where possible.

Summary measures of population health (SMPH) which combine morbidity and mortality outcomes were commonly used to estimate overall health outcomes and to calculate cost effectiveness of initiatives. SMPH are useful outcome measures as they allow comparison between different scenarios and population groups and can easily be used in cost benefit analyses. However, other outcome measures such as disease prevalence, incidence and mortality might be useful to inform decision making, and it is therefore recommended that models produce a range of outcome measures. 

Health related costs were calculated by applying the value of a statistical life (VSL) or the value of a statistical life year (VSLY) to the number of deaths and DALYs QALYs or HALYs averted, respectively. However, the VSL varied between studies, and international reviews showed wide variation in VSL between and within countries [44,45]. Further methodological development is needed in this area. Meanwhile, it is recommended that a method to cost the health benefit of active transport uses a VSL that is agreed upon by all stakeholders involved.

This review was carried out within the context of integrating the health benefits of active transport within CBAs of large infrastructure projects. Through stakeholder engagement and interviews, criteria were derived that identified important functions that a best practice model would need to fulfil. These selection criteria can be applied in other applications beyond active transport to inform model selection. As part of translation to other contexts and settings, we recommend engaging with key stakeholders to review, adapt where necessary and agree on selection criteria. Engaging stakeholders closely in the process was an important factor in enabling a successful project outcome. We utilised a partnership approach, which involved an active cross-cluster steering group, ongoing engagement with key stakeholders to identify relevant concerns, areas of interest and organisational readiness and a responsive approach to issues that arose during the project.

Based on the evidence gathered in this review and evaluation of models against a set of criteria, a MSLT model was identified as the best method currently available for use in NSW [8].

### Strengths and Limitations

To our knowledge this is the first study to systematically review models to cost the health benefits of active transport and to assess them for use in local policy context. A strength of our study is that it has direct policy relevance. The criteria for model selection were informed by a group of key stakeholders to ensure that findings were relevant and applicable for policy makers. Our findings also have wider application within other policy making and country contexts. Policy makers can identify models that fulfil both quality and usability selection criteria that suit their purposes and local context. Our findings also have wider application within other policy making and country contexts. Policy makers can identify models that fulfil both quality and usability selection criteria that suit their purposes and local context. We recommend engaging with key stakeholders to review, adapt where necessary, and agree on selection criteria. Our study has some limitations. Some studies might have been missed due to the exclusion of non-English language papers and papers from low-income country contexts. Although we included grey as well as peer reviewed published literature, there may be models that are not reported on within the public domain. The focus on higher income country contexts means that our study may have less applicability for lower-income country contexts. 

## 5. Conclusions

This review showed that there is strong interest in quantifying and costing the health benefits of active transport internationally. Based on the evidence gathered in this study, a best practice method should consider different forms of active transport and included the related exposures: physical activity, air pollution and road transport injuries. The method should consider all health outcomes which have been causally associated with these exposures. A life table model is recommended for modelling the health impacts of active transport measures.

Future work should look at establishing a best practice model based on available methods and emerging evidence. Harmonization of methods will further help to achieve comparability across studies.

## Figures and Tables

**Table 1:** Desirable characteristics of model to cost the health benefits of active transport from stakeholder consultation and criteria derived for model evaluation.

Category	Characteristics from Stakeholder Consultation	Criteria for Evaluation
**Types of active transport**	Allows for different types of active transport characterised by duration and intensity (energy expenditure)	Considers different forms of active transport (minimum cycling and walking) Duration and intensity of active transport
**Statistical model**	Allows for modelling of subgroups	Analysis by population subgroups
	Uses robust statistical model	Dynamic model States input parameter and assumptions
	Models impacts at fine grained level (person/age group/gender) as opposed to whole of population	Models impacts at fine grained level
**Exposures relevant to active transport**	Considers all exposures relevant to active transport	Physical activity Air pollution Injury
**Health outcomes**	Considers all health outcomes with sufficiently strong epidemiological evidence and causal relationship to exposure	Physical activity: breast cancer, colon cancer, ischemic stroke, ischemic heart disease and type 2 diabetes Air pollution: ischemic stroke, ischemic heart disease, tracheal, bronchus and lung cancer, and chronic obstructive pulmonary disease Injury: morbidity and mortality by transport mode
**Outcome measures**	Considers morbidity and mortality Produces outcome measures that can be used in cost benefit analysis Considers health related economic outcomes such as health care costs, productivity costs, (in addition to health outcomes)	Morbidity Mortality Years lived with disability (YLD) Years of Life Lost (YLL) Summary measure of population health Health care costs Productivity Monetisation

**Table 2:** Characteristics of studies included in the review.

Author, Date	Country	Type of Active Transport	Statistical Model	Exposures Considered	Physical Activity Outcome Measures	Injury Outcome Measures	Air pollution Outcome Measures	Summary Outcome Measures	Discounting	Evaluation Method
**Brey et al., 2016**	Spain	Cycling	BoD *	Physical activity and injury	All-cause mortality	Bike and car accidents	N/A	Avoided deaths and monetary value of avoided deaths applying value of statistical life year, cost of road transport injury	5%	CBA *
**Brown et al., 2017**	Australia	Walking and cycling	Multi state lifetable model	Physical activity and injury	Incidence of nine obesity-related diseases modelled via effect on BMI. Ischaemic heart disease, hypertensive heart disease, ischaemic stroke, diabetes, colorectal cancer, kidney cancer, breast cancer, endometrial cancer and osteoarthritis)	Mode-specific fatalities and serious injuries	N/A	YLD (for injury only), HALY and healthcare costs	3%	CBA *
**Buekers et al., 2015**	Belgium	Walking and cycling	BoD *	Air pollution, road transport injury, physical activity	Incidence: IHD, dementia, type2 diabetes, depression, colon cancer, breast cancer. Mortality: all-cause mortality (mortality risk delayed in time), Morbidity, Morbidity costs (including treatment costs and productivity costs)	Crash risk for cycling and walking, relatively to car driving from local data	All-cause mortality due to air pollution (YLL)	DALY *, external costs, cost per km, cost benefit (YLL * x VSLY *)	No	CBA *
**Cobiac et al., 2009**	Australia	Any physical activity	Multi state lifetable model	Physical activity	Ischaemic heart disease, ischaemic stroke, type 2 diabetes, breast cancer and colon cancer. Morbidity and mortality	N/A	N/A	DALY * & QALY *	3%	CUA *
**Doorley et al., 2017**	Ireland	Cycling	BoD *	Physical activity, air pollution (PM_2.5_ *), road transport injury	Cardiovascular disease, breast cancer, colon cancer, dementia, depression and type II diabetes	Morbidity and mortality from road transport injury	Respiratory diseases, cardiovascular diseases and lung cancer. Since cardiovascular disease risk is influenced by both physical activity and pollution exposure, the impacts of the two exposures were modelled multiplicatively	YLL *, YLD *, DALY *	No	HIA *
**Genter et al., 2009**	New Zealand	Walking and cycling	BoD *	Physical activity	All-cause mortality, colon, lung, breast and all cancer, CVD, type 2 diabetes (mortality), depression (incidence)	N/A	N/A	Cost benefit per km of active transport (VSL x mortality)	No	CBA *
**Gu et al., 2016**	New York, USA	Cycling	Markov model	Physical activity, air pollution (PM_2.5_ *), road transport injury	LE * gain from physical activity considered in total cost output	QALYs * from injury considered in output	LE * gain/decrease from air pollution considered in total model	QALY *, cost per QALY *	3%	CEA *
**Holm 2012**	Copenhagen, Denmark	Cycling	BoD *	Physical activity, air pollution, road transport injury	YLL * and YLD * ischaemic heart disease, ischaemic stroke, type II diabetes, breast cancer, colon cancer	YLL * and YLD * injuries	YLL * and YLD * cardiopulmonary diseases, lung cancer	DALY *	No	HIA *
**Johansson et al., 2017**	Stockholm, Sweden	Cycling	BoD *	Air pollution	N/A	N/A.	LE * gained due to decreased mortality	Years of life gained	No	HIA *
**Kahlmeier et al., 2017**	Non-specific	Walking and cycling	BoD *	Physical activity, air pollution (PM_2.5_ *), road transport injury	All-cause mortality	All-cause mortality	All-cause mortality	Mortality and cost calculated per VSLY	5%	CBA *
**Li et al., 2014**	USA	Cycling	BoD *	Physical activity	All-cause mortality and health care costs	N/A	N/A	Reduced healthcare costs, reduced mortality cost (calculated by assigning VSLY * to reduced mortality), reduced accident cost	5%	CBA *
**Macmillan et al., 2014**	New Zealand	Cycling	System dynamics model	Physical activity, air pollution, road transport injury	All-cause mortality	Serious injury and deaths caused by a collision with a light vehicle.	Deaths, cardiovascular and respiratory, carbon monoxide, COPD * hospitalizations and restricted activity days due to PM_10_ *, cancer incidence due to benzene	Deaths, hospitalisations, restricted activity days, monetary values (net benefit, cost benefit)	No	CEA *
**Mueller et al., 2017**	Spain	Physical activity	BoD *	Physical activity, air pollution, road transport injury	Physical activity all-cause mortality for YLL * and YLD *—cardiovascular disease (CVD *), stroke, type 2 diabetes, colon cancer, breast cancer and dementia.	Road transport Traffic incidents with injuries (fatal or non-fatal)	Air pollution, all-cause mortality, cardiovascular disease (CVD *), stroke, type 2 diabetes, respiratory hospital admissions, preterm birth, low birth weight.	YLL *, YLD *, DALY *	No	HIA *
**Rojas-Rueda et al., 2013**	Spain	Cycling	BoD *	Physical activity, air pollution, road transport injury	Physical activity: cardiovascular disease, type 2 diabetes, breast cancer, colon cancer, dementia	Road traffic incidents: minor and major injuries	Air pollution: CVD *, cerebrovascular disease, lower respiratory tract infection, low birth weight and preterm birth	Morbidity and DALY *	No	HIA *
**Saelensminde 2004**	Norway	Walking and cycling	Other-cost savings per new active traveller	Physical activity	The four types of diseases are cancer (five different types), high blood pressure, type 2 diabetes and musculoskeletal ailments.	N/A	N/A	Cost	3% and 8%	CBA *
**Stokes et al., 2007**	US	Walking	Other cost saving by applying cost of obesity from other study	Physical activity	Obesity and obesity related costs	N/A	N/A	Cost	No	CBA *
**Taddei et al., 2014**	Italy	Cycling	BoD *	Physical activity, road transport injury	All-cause mortality, incidence type 2 diabetes, AMI *, heart failure, stroke	Road traffic accidents and fatalities by mode of transport	N/A	Incidence, mortality, treatment cost and cost	5%	CEA *
**Woodcock, et al., 2013**	UK	Physical activity	BoD *	Physical activity, air pollution, road transport injury	CVD *, colon cancer, breast cancer, diabetes, dementia, depression, all-cause mortality	Road transport injury	Cardio-respiratory diseases, lung cancer, acute respiratory infections	DALY *	No	HIA *
**Zapata-Diomedi et al., 2017**	Australia	Walking and cycling	Multi state lifetable model	Physical activity, air pollution, road transport injury	Breast cancer, colon cancer, ischemic Stroke, ischemic heart disease, type 2 diabetes	Road transport injury	Ischemic stroke, ischemic heart disease, tracheal, bronchus and lung cancer, COPD *	Health care costs, life years, HALYs *, prevalent cases, deaths, YLD *	No	CBA *
**Zheng et al., 2010**	Australia	Walking	BoD *	Physical activity	CHD *	N/A	N/A	Health care cost saving	No	CBA *

* BoD, Burden of Disease; CVD, cardio vascular disease; CBA, cost benefit analysis; CUA, cost utility analysis; CEA, cost effectiveness analysis; HIA, health impact assessment; COPD, chronic obstructive pulmonary disease; DALY, disability adjusted life year; YLL, years of life lost; YLD, years lived with disability; QALY, quality adjusted life year; LE, life expectancy; HALY, health adjusted life year; PM_2.5_, particulate matter ≤ 2.5 μm; PM_10_, particulate matter ≤ 10 μm; AMI, acute myocardial infarction.

**Table 3:** Evaluation of studies against criteria derived during stakeholder consultation.

Criteria	Brey et al., 2016	Brown et al., 2017	Buekers et al., 2015	Cobiac et al., 2009	Doorley et al., 2017	Genter et al., 2009	Gu et al., 2016	Holm 2012	Kahlmeier et al., 2017	Macmillan et al., 2014	Mueller et al., 2017	Rojas-Rueda et al., 2013	Taddei et al., 2014	Woodcock et al., 2013	Zapata-Diomedi et al., 2017	Zheng et al., 2010
**Active Transport Modes**																
**Different forms of active transport (minimum cycling and walking)**	No	Yes	Yes	Yes	Yes	Yes	No	No	Yes	No	Yes	Yes	No	Yes	Yes	No
**Duration and intensity of active transport**	No	Yes	No	Yes	Yes	No	Yes	No	Yes	Yes	Yes	Yes	No	Yes	Yes	No
**Exposures Relevant to Active Transport**																
**Physical activity**	Yes	Yes	Yes	Yes	Yes	Yes	Yes	Yes	Yes	Yes	Yes	Yes	Yes	Yes	Yes	Yes
**Air pollution**	No	No	Yes	No	Yes	No	Yes	Yes	Yes	Yes	Yes	Yes	No	Yes	Yes	No
**Injury**	Yes	Yes	Yes	No	Yes	No	Yes	Yes	Yes	Yes	Yes	Yes	Yes	Yes	Yes	No
**Statistical Model**																
**States input parameter and assumptions**	No	Yes	Yes	Yes	Yes	Yes	Yes	No	Yes	Yes	Yes	Yes	Yes	Yes	Yes	Yes
**Analysis by population subgroups**	Yes *	Yes *	Yes *	Yes *	Yes *	No	Yes *	Yes *	Yes *	Yes *	Yes *	Yes *	Yes *	Yes *	Yes *	No
**Dynamic model**	No	Yes	No	Yes	No	No	Yes	No	No	Yes	No	No	No	No	Yes	No
**Models at fine grained level**	No	Yes	No	Yes	Yes	No	Yes	Yes	No	Yes	Yes	No	No	Yes	Yes	No
**Heath Outcomes (Minimum Included) ^#^**																
**Physical activity**	No	Obesity related outcomes	Yes	Yes	Yes	Yes	No	Yes	No	No	Yes	Yes	Yes	Yes	Yes	No
**Air pollution**	No	No	No	No	Yes	No	No)	No	No	Yes	Yes	Yes	No	Yes	Yes	No
**Injury**	Yes	Yes	Yes	No	Yes	No	No	Yes	No	Yes	Yes	Yes	Yes	Yes	Yes	No
**Outcome Measures**																
**Morbidity**	No	No	No	No	No	No	No	No	No	Yes	No	Yes	Yes	No	Yes	Yes
**Mortality**	Yes	Yes	No	No	No	No	Yes	No	Yes	Yes	No	No	Yes	No	Yes	No
**YLD**	No	Yes	Yes	Yes	Yes	No	No	Yes	No	No	Yes	Yes	No	Yes	Yes	No
**YLL**	No	No	Yes	Yes	Yes	No	No	Yes	No	No	Yes	Yes	No	Yes	Yes	No
**Summary measure of population health**	No	Yes	Yes	Yes	Yes	Yes	Yes	Yes	No	No	Yes	Yes	No	Yes	Yes	No
**Health care costs**	Yes	Yes	No	No	No	Yes	No	No	No	No	No	No	No	No	Yes	Yes
**Productivity**	No	No	No	No	No	No	No	No	No	No	No	No	Yes	No	Yes (Later Model)	
**Monetisation**	Yes	Yes	Yes	No	No	Yes	Yes	No	Yes	Yes	No	No	No	No	Yes	Yes

* If modelled separately. ^#^ Minimum outcomes to be included: Physical activity: Breast cancer, colon cancer, ischemic Stroke, ischemic heart disease and type 2 diabetes. Air pollution: Ischemic Stroke, Ischemic heart disease, tracheal, bronchus and lung cancer, COPD. Injury: Morbidity and mortality.

## Supplementary Materials

The following are available online at https://www.mdpi.com/1660-4601/17/17/6186/s1, Table S1: Search strategy to identify studies that present methods to cost the health benefits of active transport, Table S2: Database search, search algorithm, restrictions and number of records identified, Table S3: Systematic review data extraction fields, Table S4: Overview of CHEERS guideline items reported in the included cost-effectiveness studies, Table S5: Studies excluded after full text analysis. Box S1: Burden of Disease and Multistate Life Table methods.
