# Supplementary material for: What Is the Best Practice Method for Quantifying the Health and Economic Benefits of Active Transport?
Source: Int J Environ Res Public Health. 2020 Aug 26;17(17):6186. doi: 10.3390/ijerph17176186 (PMC7503465; doi:10.3390/ijerph17176186)
Supplement: Supplementary file 1 [file ijerph-17-06186-s001.pdf]

Table S1. Search strategy to identify studies that present methods to cost the health benefits of active transport.

**1. Transport/ and urban form search terms**<br>"active transport" OR "active travel" OR "non-mechanised transport*" OR "non-mechanized transport*" OR "active mobility" OR "non-motorised transport*" OR "non-motorized transport*" OR "human powered transport*" OR walk* OR pedestrian* OR bike* OR bicycle* OR bicycling OR cyclist OR "active living" OR "travel mode" OR "public transport*" OR bus OR buses OR railway OR railways OR railroad OR railroads OR "light rail" OR "public transport" OR "sustainable transport" OR "built environment" OR "urban planning" OR "urban design" OR cycling OR rail OR "healthy built environment" OR "healthy planning" OR liveability OR liveable OR livable OR livability OR train
**2. Economic and health search terms**<br>"economic benefit*" OR "economic model*" OR "cost benefit*" OR "cost saving*" OR "economic impact*" OR "economic evaluation*" OR "cost utility analysis" OR "cost utility tool*" OR "cost effective*" OR "money saved" OR "health economic assessment tool*" OR "health impact*" OR "health implication*" OR "cost effective*" OR "health effect*" OR "health benefit*" OR "health outcome*" OR "health improvement*" OR "life* saved" OR "life expectancy" OR "risk-benefit assessment" OR "health gain " OR "cost consequence" OR "economic appraisal"
**3. Search strategy**<br>**#1 AND #2**

Table S2. Database search, search algorithm, restrictions and number of records identified.

Database	Search algorithm	Restrictions	Number of records	Date of search
EBSCO Host (Cinhal, Business Source Premier, Sport Discus)	(TI ("active transport" OR "active travel" OR "non-mechanised transport*" OR "non-mechanized transport*" OR "active mobility" OR "non-motorised transport*" OR "non-motorized transport*" OR "human powered transport*" OR walk* OR pedestrian* OR bike* OR bicycle* OR bicycling OR cyclist OR "active living" OR "travel mode" OR "public transport*" OR bus OR buses OR railway OR railways OR railroad OR railroads OR "light rail" OR "public transport" OR "sustainable transport" OR "built environment" OR "urban planning" OR "urban design" OR cycling OR rail OR "healthy built environment" OR "healthy planning" OR liveability OR liveable OR livable OR livability OR train) AND TI ("economic benefit*" OR "economic model*" OR "cost benefit*" OR "cost saving*" OR "economic impact*" OR "economic evaluation*" OR "cost utility analysis" OR "cost utility tool*" OR "cost effective*" OR "money saved" OR "health economic assessment tool*" OR "health impact*" OR "health implication*" OR "cost effective*" OR "health effect*" OR "health benefit*" OR "health outcome*" OR "health improvement*" OR "life* saved" OR "life expectancy" OR "risk-benefit assessment" OR "health gain" OR "cost consequence" OR "economic appraisal"))	2000 to current, English language, journals, books, dissertations, title only	208	20/05/2019
OVID – (Cochrane database of systematic reviews, NHS economic evaluation database, Embase, Emcare, PsycInfo)	("active transport" or "active travel" or "non-mechanised transport*" or "non-mechanized transport*" or "active mobility" or "non-motorised transport*" or "non-motorized transport*" or "human powered transport*" or walk* or pedestrian* or bike* or bicycle* or bicycling or cyclist or "active living" or "travel mode" or "public transport*" or bus or buses or railway or railways or railroad or railroads or "light rail" or "public transport" or "sustainable transport" or "built environment" or "urban planning" or "urban design" or cycling or rail or "healthy built environment" or "healthy planning" or "liveability" or "liveable" or "livable" or "livability" or " train").mp. and ("economic benefit*" or "economic model*" or "cost benefit*" or "cost saving*" or "economic impact*" or "economic evaluation*" or "cost utility analysis" or "cost utility tool*" or "cost effective*" or "money saved" or "health economic assessment tool*" or "health impact*" or "health implication*" or "cost effective*" or "health effect*" or "health benefit*" or "health outcome*" or "health improvement*" or "life* saved" or "life expectancy" or "risk-benefit assessment" or "health gain" or "cost consequence" or "economic appraisal").m_titl. [mp=ti, ot, ab, tx, kw, ct, sh, hw, tn, dm, mf, dv, fx, dq]	2000 to current, title only, English language	1107	20/05/2019
GeoBase & Georef	found in GEOBASE for 2000-2020: ((("active transport" OR "active travel" OR "non-mechanised transport*" OR "non-mechanized transport*" OR "active mobility" OR "non-motorised transport*" OR "non-motorized transport*" OR "human powered transport*" OR walk* OR pedestrian* OR bike* OR bicycle* OR bicycling OR cyclist OR "active living" OR "travel mode" OR "public transport*" OR bus OR buses OR railway OR railways OR railroad OR railroads OR "light rail" OR "public transport" OR "sustainable transport" OR "built environment" OR "urban planning" OR "urban design" OR cycling OR rail OR "healthy built environment" OR "healthy planning" OR liveability OR liveable OR livable OR livability OR train) AND ("economic benefit*" OR "economic model*" OR "cost benefit*" OR "cost saving*" OR "economic impact*" OR "economic evaluation*" OR "cost utility analysis" OR "cost utility tool*" OR "cost effective*" OR "money saved" OR "health economic assessment tool*" OR "health impact*" OR "health implication*" OR "cost effective*" OR "health effect*" OR "health benefit*" OR	2000 to current , English, title only	80	20/05/2019

Database	Search algorithm	Restrictions	Number of records	Date of search
	"health outcome*" OR "health improvement*" OR "life* saved" OR "life expectancy" OR "risk-benefit assessment" OR "health gain")) WN TI), English only			
PubMed	Search (((("economic benefit*"[Title/Abstract] OR "economic model*"[Title/Abstract] OR "cost benefit*"[Title/Abstract] OR "cost saving*"[Title/Abstract] OR "economic impact*"[Title/Abstract] OR "economic evaluation*"[Title/Abstract] OR "cost utility analysis"[Title/Abstract] OR "cost utility tool*"[Title/Abstract] OR "cost effective*"[Title/Abstract] OR "money saved"[Title/Abstract] OR "health economic assessment tool*"[Title/Abstract] OR "health impact*"[Title/Abstract] OR "health implication*"[Title/Abstract] OR "cost effective*"[Title/Abstract] OR "health effect*"[Title/Abstract] OR "health benefit*"[Title/Abstract] OR "health outcome*"[Title/Abstract] OR "health improvement*"[Title/Abstract] OR "life* saved"[Title/Abstract] OR "life expectancy"[Title/Abstract] OR "risk-benefit assessment"[Title/Abstract] OR "health gain "[Title/Abstract] OR "cost consequence"[Title/Abstract] OR "economic appraisal"[Title/Abstract])) AND ("2000/01/01"[PDat] : "2020/12/31"[PDat]) AND Humans[Mesh] AND English[lang])) AND ((("active transport"[Title/Abstract] OR "active travel"[Title/Abstract] OR "non-mechanised transport*"[Title/Abstract] OR "non-mechanized transport*"[Title/Abstract] OR "active mobility"[Title/Abstract] OR "non-motorised transport*"[Title/Abstract] OR "non-motorized transport*"[Title/Abstract] OR "human powered transport*"[Title/Abstract] OR walk*[Title/Abstract] OR pedestrian*[Title/Abstract] OR bike*[Title/Abstract] OR bicycle*[Title/Abstract] OR bicycling[Title/Abstract] OR cyclist[Title/Abstract] OR "active living"[Title/Abstract] OR "travel mode"[Title/Abstract] OR "public transport*"[Title/Abstract] OR bus[Title/Abstract] OR buses[Title/Abstract] OR railway[Title/Abstract] OR railways[Title/Abstract] OR railroad[Title/Abstract] OR railroads[Title/Abstract] OR "light rail"[Title/Abstract] OR "public transport"[Title/Abstract] OR "sustainable transport"[Title/Abstract] OR "built environment"[Title/Abstract] OR "urban planning"[Title/Abstract] OR "urban design"[Title/Abstract] OR cycling[Title/Abstract] OR rail[Title/Abstract] OR "healthy built environment"[Title/Abstract] OR "healthy planning"[Title/Abstract] OR liveability[Title/Abstract] OR liveable[Title/Abstract] OR livable[Title/Abstract] OR livability[Title/Abstract] OR train[Title/Abstract])) AND ("2000/01/01"[PDat] : "2020/12/31"[PDat]) AND Humans[Mesh] AND English[lang]) Filters: Publication date from 2000/01/01 to 2020/12/31; Humans; English	English language, published 2000 to current, humans, title, abstract	1075	20/05/2019
Scopus	(SUBJAREA (medi OR nurs OR vete OR dent OR heal OR mult) SUBJAREA (arts OR busi OR deci OR econ OR psyc OR soci) TITLE-ABS-KEY ("active transport" OR "active travel" OR "non-mechanised transport*" OR "non-mechanized transport*" OR "active mobility" OR "non-motorised transport*" OR "non-motorized transport*" OR "human powered transport*" OR walk* OR pedestrian* OR bike* OR bicycle* OR bicycling OR cyclist OR "active living" OR "travel mode" OR "public transport*" OR bus OR buses OR railway OR railways OR railroad OR railroads OR "light rail" OR "public transport" OR "sustainable transport" OR "built environment" OR "urban planning" OR "urban design" OR cycling OR rail OR "healthy built environment" OR "healthy planning" OR liveability OR liveable OR livable OR livability OR train) AND TITLE-ABS-KEY ("economic benefit*" OR "economic model*" OR "cost benefit*" OR "cost saving*" OR "economic impact*" OR "economic evaluation*" OR "cost utility analysis" OR "cost utility tool*" OR "cost effective*" OR "money saved" OR "health economic assessment tool*" OR "health impact*" OR "health implication*" OR "cost	Subject areas social science and medicine, English language, published 2005-2019, title, abstract. key word	640	20/05/2019

Database	Search algorithm	Restrictions	Number of records	Date of search
	effective*" OR "health effect*" OR "health benefit*" OR "health outcome*" OR "health improvement*" OR "life* saved" OR "life expectancy" OR "risk-benefit assessment" OR "health gain " OR "cost consequence" OR "economic appraisal")) AND PUBYEAR > 1999 AND (LIMIT-TO (LANGUAGE,"English"))			
Web of Science	(TI = ("active transport" OR "active travel" OR "non-mechanised transport*" OR "non-mechanized transport*" OR "active mobility" OR "non-motorised transport*" OR "non-motorized transport*" OR "human powered transport*" OR walk* OR pedestrian* OR bike* OR bicycle* OR bicycling OR cyclist OR "active living" OR "travel mode" OR "public transport*" OR bus OR buses OR railway OR railways OR railroad OR railroads OR "light rail" OR "public transport" OR "sustainable transport" OR "built environment" OR "urban planning" OR "urban design" OR cycling OR rail OR "healthy built environment" OR "healthy planning" OR liveability OR liveable OR livable OR livability OR train) AND TI=("economic benefit*" OR "economic model*" OR "cost benefit*" OR "cost saving*" OR "economic impact*" OR "economic evaluation*" OR "cost utility analysis" OR "cost utility tool*" OR "cost effective*" OR "money saved" OR "health economic assessment tool*" OR "health impact*" OR "health implication*" OR "cost effective*" OR "health effect*" OR "health benefit*" OR "health outcome*" OR "health improvement*" OR "life* saved" OR "life expectancy" OR "risk-benefit assessment" OR "health gain" OR "cost consequence" OR "economic appraisal")) AND LANGUAGE: (English) AND DOCUMENT TYPES: (Article) Indexes=SCI-EXPANDED, SSCI, A&HCI, CPCI-S, BKCI-SSH, ESCI Timespan=2000-2019	English, Published 2000-2019, title only	566	20/05/2019
Proquest ABI/IFORM	ti(("active transport" OR "active travel" OR "non-mechanised transport*" OR "non-mechanized transport*" OR "active mobility" OR "non-motorised transport*" OR "non-motorized transport*" OR "human powered transport*" OR walk* OR pedestrian* OR bike* OR bicycle* OR bicycling OR cyclist OR "active living" OR "travel mode" OR "public transport*" OR bus OR buses OR railway OR railways OR railroad OR railroads OR "light rail" OR "public transport" OR "sustainable transport" OR "built environment" OR "urban planning" OR "urban design" OR cycling OR rail OR "healthy built environment" OR "healthy planning" OR liveability OR liveable OR livable OR livability OR train) AND ("economic benefit*" OR "economic model*" OR "cost benefit*" OR "cost saving*" OR "economic impact*" OR "economic evaluation*" OR "cost utility analysis" OR "cost utility tool*" OR "cost effective*" OR "money saved" OR "health economic assessment tool*" OR "health impact*" OR "health implication*" OR "cost effective*" OR "health effect*" OR "health benefit*" OR "health outcome*" OR "health improvement*" OR "life* saved" OR "life expectancy" OR "risk-benefit assessment" OR "health gain" OR "cost consequence" OR "economic appraisal"))	English, 2000-2019, peer reviewed, title	246	20/05/2019

Table S3. Systematic review data extraction fields.

Author, date
Country
Study objective / aim
Type of active transport
Baseline active transport use and scenarios for change in travel patterns
Statistical model
Data requirements / model input parameters
Model assumptions, sensitivity analysis, analysis by population subgroups
Exposures considered
Physical activity outcomes
Injury outcomes
Air pollution outcomes
Outcome measures
Discount rate
Economic evaluation method

Table S4. Studies excluded after full text analysis.

Study	Reason for exclusion
**Australian Transport and Infrastructure Council (ATAP) (2016)[1]**	Uses same methodology as Genter (2009)
**Beale et al (2012)[2]**	Model is not reproducible from study
**Beavis and Moodie (2014)[3]**	Model is not reproducible from study
**Cavill et al (2007)[4]**	Review
**Chapman et al. (2018)[5]**	Application of ITHIM model Woodcock (2013)
**Deenihan and Caulfield (2014)[6]**	HEAT application
**Elvik (2000)[7]**	Review
**Fishman (2015)[8]**	Application of HEAT
**Gotschi (2011)[9]**	HEAT application
**Gunn et al. (2014)[10]**	Does not state if and how health benefits were considered focusses mainly on exposure - i.e. change in walking behaviour
**Guo et al. (2010)[11]***	Does not state how health was considered in model / not specific to active transport
**Hankey (2012)[12]**	Focus on built environment, no economic evaluation
**Knell et al. (2019)[13]**	Cohort study not transferrable method
**Kwan et al. (2017)[14]**	Application of ITHIM model Woodcock (2013) / no cost benefit analysis
**Li & Faghri (2014)[15]**	HEAT application
**Lindsay (2011)[16]**	Application of HEAT, not a cost benefit analysis
**Mansfield & Gibson (2015)[17]**	No cost benefit analysis / not active transport specific only physical activity
**Moodie et al. (2009)[18]**	Model only for active school bus and obesity / walking to school not for other exposures / outcomes
**Moodie et al (2011)[19]**	Model only for active school bus and obesity / walking to school not for other exposures / outcomes
**Mueller (2016)[20]**	No cost benefit analysis and not active transport specific
**Mueller (2018)[21]**	Application of UTHOPIA tool included in Mueller (2017) publication
**Mueller (2018)[22]**	Application of UTHOPIA tool included in Mueller (2017) publication
**Mulley et al. (2012)[23]**	Not clear how health benefits were calculated from paper - paper does not show methodology
**Perez et al. (2017)[24]**	Application of HEAT
**Queensland Department of Transport and Main Roads (2016)**	Same methodology as Genter (2009)
**Rabl & Nazelle (2011)[25]**	Application of HEAT
**Rojas-Rueda et al. (2016)[26]**	Same model as Rojas-Rueda (2013)
**Rojas-Rueda et al (2012)[27]**	Same model as Rojas-Rueda (2013), but life expectancy and mortality as outcome measures instead of combined measure of DALY
**Rutter et al (2013)[28]**	Heat tool, more up to date publication included, Kahlmeier (2017)
**Sa et al. (2017)[29]**	Application of ITHIM model Woodcock / no cost benefit analysis

**Tainio et al. 2017**[30]	Application of ITHIM model Woodcock / no cost benefit analysis
**Tas et al. 2019**[31]	HEAT application
**Woodcock 2009**[32]	Application of ITHIM model Woodcock (2013) / no cost benefit analysis
**Zapata-Diomedi et al. 2018**[33]	Same model as Zapata-Diomedi (2017) which is included
**Zapata-Diomedi et al. 2019**[34]	Same model as Zapata-Diomedi (2017) which is included

Box S1: Burden of Disease and Multistate Life Table methods.

Burden of Disease (BoD) methodology

The BoD methodology has been used in the Global Burden of Disease study to quantify the burden of disease attributable to risk factors.[36] It uses the mathematical concept of the population attributable fraction (PAF). The PAF is the proportion of cases for an outcome of interest that can be attributed to a given risk factor among the entire population.[37] Input parameters to calculate the PAF are the exposure of the population to the risk factor and the strength of the association between exposure and health outcome. The burden of disease that could be avoided through elimination of a risk factor, can then be calculated by multiplying the PAF by the number of observed and predicted cases for the associated health outcomes in the population.

Multistate Life Table methods

The proportional multistate life table (MSLT) method calculates changes in health outcomes from uptake of active transport by simulating two populations, the population as it is (or is expected to be in future years), and an identical population that has been exposed to changes in active transport.[38] Compared with a traditional life table, the proportional MSLT allows dividing those alive into healthy and diseased and allows for the possibility of including multiple diseases and comorbidities. The effect of active

References

1. Australian Transport Assessment and Planning (ATAP). Australian Transport Asessment and Planning Guidelines M4 Active Travel; 2015.
2. Beale SJ, Bending MW, Trueman P, Naidoo B. Should we invest in environmental interventions to encourage physical activity in England? An economic appraisal. *European journal of public health* 2012; **22**(6): 869-73.
3. Beavis MJ, Moodie M. Incidental physical activity in Melbourne, Australia: health and economic impacts of mode of transport and suburban location. *Health promotion journal of Australia : official journal of Australian Association of Health Promotion Professionals* 2014; **25**(3): 174-81.
4. Cavill N, Kahlmeier S, Rutter H, Racioppi F, Oja P. Economic assessment of transport infrastructure and policies. Methodological guidance on the economic appraisal of health effects related to walking and cycling. 2007.
5. Chapman R, Keall M, Howden-Chapman P, et al. A Cost Benefit Analysis of an Active Travel Intervention with Health and Carbon Emission Reduction Benefits. *International journal of environmental research and public health* 2018; **15**(5).
6. Deenihan G, Caulfield B. Estimating the health economic benefits of cycling. *J Transp Health* 2014; **1**(2): 141-9.
7. Elvik R. Which are the relevant costs and benefits of road safety measures designed for pedestrians and cyclists? *Accident; analysis and prevention* 2000; **32**(1): 37-45.
8. Fishman E, Schepers P, Maria Kamphuis CB. Dutch Cycling: Quantifying the Health and Related Economic Benefits. *American journal of public health* 2015; **105**(8): e13-e5.
9. Gotschi T. Costs and benefits of bicycling investments in Portland, Oregon. *Journal of physical activity & health* 2011; **8 Suppl 1**: S49-58.
10. Gunn LD, Lee Y, Geelhoed E, Shiell A, Giles-Corti B. The cost-effectiveness of installing sidewalks to increase levels of transport-walking and health. *Preventive medicine* 2014; **67**: 322-9.
11. Guo JY, Gandavarapu SJPm. An economic evaluation of health-promotive built environment changes. 2010; **50**: S44-S9.

12. Hankey S, Marshall JD, Brauer M. Health Impacts of the Built Environment: Within-Urban Variability in Physical Inactivity, Air Pollution, and Ischemic Heart Disease Mortality. *Environmental health perspectives* 2012; **120**(2): 247-53.
13. Knell G, Brown HS, Gabriel KP, et al. Cost-Effectiveness of Improvements to the Built Environment Intended to Increase Physical Activity. *Journal of physical activity & health* 2019; **16**(5): 308-17.
14. Kwan SC, Tainio M, Woodcock J, Sutan R, Hashim JH. The carbon savings and health co-benefits from the introduction of mass rapid transit system in Greater Kuala Lumpur, Malaysia. *J Transp Health* 2017; **6**: 187-200.
15. Li M, Faghri A. Cost-Benefit Analysis of Added Cycling Facilities. *Transportation Research Record* 2014; (2468): 55-63.
16. Lindsay G, Macmillan A, Woodward A. Moving urban trips from cars to bicycles: Impact on health and emissions; 2011.
17. Mansfield TJ, Gibson JM. Health Impacts of Increased Physical Activity from Changes in Transportation Infrastructure: Quantitative Estimates for Three Communities. *BioMed Research International* 2015; **2015 (no pagination)**(812325).
18. Moodie M, Haby M, Galvin L, Swinburn B, Carter RJIjobn, activity p. Cost-effectiveness of active transport for primary school children-Walking School Bus program. 2009; **6**(1): 63.
19. Moodie M, Haby MM, Swinburn B, Carter R. Assessing cost-effectiveness in obesity: active transport program for primary school children--TravelSMART Schools Curriculum program. *Journal of physical activity & health* 2011; **8**(4): 503-15.
20. Mueller N, Rojas-Rueda D, Basagaña X, et al. Urban and Transport Planning Related Exposures and Mortality: A Health Impact Assessment for Cities; 2016.
21. Mueller N, Rojas-Rueda D, Khreis H, et al. Socioeconomic inequalities in urban and transport planning related exposures and mortality: A health impact assessment study for Bradford, UK. *Environment international* 2018; **121**(Pt 1): 931-41.
22. Mueller N, Rojas-Rueda D, Salmon M, et al. Health impact assessment of cycling network expansions in European cities. *Preventive medicine* 2018; **109**: 62-70.
23. Mulley C, Tyson R, McCue P, Rissel C, Munro C. Valuing active travel: Including the health benefits of sustainable transport in transportation appraisal frameworks. *Research in Transportation Business and Management* 2013; **7**: 27-34.
24. Pérez K, Olabarria M, Rojas-Rueda D, Santamariña-Rubio E, Borrell C, Nieuwenhuijsen M. The health and economic benefits of active transport policies in Barcelona. *J Transp Health* 2017; **4**: 316-24.
25. Rabl A, De Nazelle AJTp. Benefits of shift from car to active transport. 2012; **19**(1): 121-31.
26. Rojas-Rueda D, De Nazelle A, Andersen ZJ, et al. Health impacts of active transportation in Europe. 2016; **11**(3): e0149990.
27. Rojas-Rueda D, de Nazelle A, Teixido O, Nieuwenhuijsen MJ. Replacing car trips by increasing bike and public transport in the greater Barcelona metropolitan area: a health impact assessment study. *Environment international* 2012; **49**: 100-9.
28. Rutter H, Cavill N, Racioppi F, Dinsdale H, Oja P, Kahlmeier S. Economic impact of reduced mortality due to increased cycling. *American journal of preventive medicine* 2013; **44**(1): 89-92.
29. Sa TH, Tainio M, Goodman A, et al. Health impact modelling of different travel patterns on physical activity, air pollution and road injuries for Sao Paulo, Brazil. *Environment international* 2017; **108**: 22-31.
30. Tainio M, Monsivais P, Jones NR, Brand C, Woodcock J. Mortality, greenhouse gas emissions and consumer cost impacts of combined diet and physical activity scenarios: a health impact assessment study. *BMJ open* 2017; **7**(2): e014199.
31. Tas A, Karagulle D, Ozcan S, Ek HN, Kiraz EDE. Evaluation of walking level health and economic benefits by europe health economic assessment tool for walking (heat - for walking). *Journal of Environmental Protection and Ecology* 2019; **20**(1): 461-7.
32. Woodcock J, Edwards P, Tonne C, et al. Public health benefits of strategies to reduce greenhouse-gas emissions: urban land transport. *Lancet (London, England)* 2009; **374**(9705): 1930-43.
33. Zapata-Diomedi B, Gunn L, Giles-Corti B, Shiell A, Lennert Veerman J. A method for the inclusion of physical activity-related health benefits in cost-benefit analysis of built environment initiatives. *Preventive medicine* 2018; **106**: 224-30.

34. Zapata-Diomedi Bn, Giles-Corti B, Claire B, et al. Physical activity-related health and economic benefits of building walkable neighbourhoods: a modelled comparison between brownfield and greenfield developments. *international journal of behavioral nutrition and physical activity* 2019; **16**(1): 11-p. .
